# The undiagnosed potential clinically significant incidental findings of neck CTA: A large retrospective single-center study

**DOI:** 10.1097/MD.0000000000022440

**Published:** 2020-10-23

**Authors:** Guangliang Chen, Yunjing Xue, Jin Wei, Qing Duan

**Affiliations:** a Department of Radiology, Fujian Medical University Union Hospital; b School of Medical Technology and Engineering, Fujian Medical University, University Town, Fuzhou, China.

**Keywords:** computed tomographic, incidental findings, CT angiography, neck

## Abstract

To assess the prevalence and missed reporting rate of potential clinically-significant incidental findings (IFs) in the neck CTA scans.

All consecutive patients undergoing neck CTA imaging, from January 1, 2017 to December 31, 2018, were retrospectively evaluated by a radiologist for the presence of incidental findings in the upper chest, lower head and neck regions. These incidental findings were subsequently classified into 3 categories in terms of clinical significance: Type I, highly significant, Type II, moderately significant; and Type III, mildly or not significant. Type I and Type II IFs were determined as potential clinically significant ones and were retrospectively analyzed by another 2 radiologists in consensus. The undiagnosed findings were designated as those that were not reported by the initial radiologists. The differences in the rate of unreported potential clinically significant IFs were compared between the chest group and head or neck group.

A total of 376 potential clinically significant IFs were detected in 1,698 (91.19%) patients, of which 175 IFs were classified as highly significant findings (Type I), and 201 (53.46%) as moderately significant findings (Type II). The most common potential clinically significant findings included thyroid nodules (n = 88, 23.40%), pulmonary nodules (n = 56, 14.89%), sinus disease (n = 39, 10.37%), intracranial or cervical artery aneurysms (n = 30, 7.98%), enlarged lymph nodes (n = 24, 6.38%), and pulmonary embolism (n = 19, 5.05%). In addition, 184 (48.94%) of them were not mentioned in the initial report. The highest incidence of missed potential clinical findings were pulmonary embolism and pathologic fractures and erosions (100% for both). The unreported rate of the chest group was significantly higher than that of the head or neck one, regardless of Type I, Type II or all potential clinically significant IFs (χ^2^ = 32.151, χ^2^ = 31.211, χ^2^ = 65.286, respectively; *P* < .001 for all).

Important clinically significant incidental findings are commonly found in a proportion of patients undergoing neck CTA, in which nearly half of these patients have had potential clinically significant IFs not diagnosed in the initial report. Therefore, radiologists should beware of the importance of and the necessity to identify incidental findings in neck CTA scans.

## 1 Introduction

With the rapid technological advance in image acquisition and spatial resolution, computed tomographic angiography (CTA) serves increasingly as a quick, accurate, and attractive noninvasive clinical procedure^[1,2]^ and has largely replaced digital subtraction angiography (DSA). The latter has long been considered as standard diagnostic procedure, an imaging modality for assessing the vessels of lower extremity,^[3]^ cardiac,^[4]^ pulmonary artery.^[5]^

In addition to diagnosing the target vessel, CTA can detect a trail of incidental findings (IFs) within the anatomic coverage. Incidental finding refers to an observed abnormality on the images that is not related to the initial indication for the examination. Previous studies have assessed the incidence and significance of incidental findings detected on CTA of the thorax aorta,^[6]^ upper extremity^[7]^ and pulmonary arterial system.^[8,9]^ Although some incidental findings, such as the degeneration of skeleton system, may have little clinical significance, the detection of others, such as early detection of curable tumors, may mean a huge difference to the patients.

Indications for neck CTA include acute ischemic stroke, vascular stenosis, etc., and the scanning coverage extends from the aortic arch to the frontal sinus, so the incidental findings of the cerebral parenchyma, soft tissues and skeleton of the head and neck, and upper thorax could be obtained. However, indications for neck CTA may vary from person to person in the patient population, which may influence the missed-report rate and incidence of incidental findings. This reality may account for the scarcity of related research in the literature. To our best knowledge, little evidence is available to probe into the missed reporting rate of incidental findings on neck CTA. A retrospective study by Kanesa-Thasan R et al^[10]^ evaluating vascular and other incidental findings on head and neck CTA, recorded clinically significant findings in 21.5% of cases. But the enrolled case size was not large enough and the issue of underreported IFs was spared.

The current study attempted to assess the prevalence and the missed reporting rate of potential clinically significant IFs during neck CTA examination and to evaluate the reasons that may influence their omission by radiologists. The study will help radiologists to avoid misinterpretations and clinicians in their clinical decision-making process.

## 2 Materials and methods

This study was approved by the Ethics Committee of Fujian Medical University Union Hospital, and the stipulation for informed consent was waived.

### 2.1 Patient selection

One radiologist with 9 years of experience, blinded to the initial CT reports, retrospectively assessed all neck CTA studies, between January 1, 2017 and December 31, 2018, only for detecting IFs. All cases were distinguished by searching our PACS (YLZ Healthcare) between the above-mentioned dates using the keyword “neck CTA.” Participants were excluded in our study if there was substantial artifact or the images were not retrievable. For patients receiving repeated neck CTA during the study period, only the first examination was included in the study. In the remaining cases, if one or more incidental findings were found in a case, it was recorded as a “potential IF” case.

### 2.2 Computed tomography technique

All examinations were performed on one of two 64-multidetector CT (MDCT) scanners (GE Healthcare, Milwaukee, Wisconsin) at our imaging center, utilizing test bolus software to acquire the optimal trigger time of CTA. FOV spanned from the aortic arch to the frontal sinus. On the basis of the patient's weight, a dose of 40 to 80 ml of nonionic iodinated contrast media (350 mg iodine/ml; Omnipaque 350, GE Healthcare) and 20 to 30 mL saline chaser were injected via an antecubital vein through an 18-gauge peripheral intravenous line at a flow rate of 4 to 5 ml/s with a power injector. Scan parameters included 120 kVp, auto mAs, and slice thickness of 0.6 to 1 mm.

### 2.3 Image analysis

Axial images were first analyzed and coronal and sagittal multi-planar reformation (MPR) were also reviewed if necessary. Images were assessed on a PACS (YLZ Healthcare) work-station. The images were evaluated with windows and levels suitable for the structures under consideration. For this reason, lung scans were evaluated using a window both for the mediastinum (width 350; level 30) and for the lung parenchyma (width 1,500; level 550), whereas for scans of head and neck, a window and level suitable for evaluating both parenchymatous organs (width 420; level 50) and bone structures (width 1,800; level 400) was employed. A window fit for vessels (width 900; level 100) was utilized to evaluate vessel IFs.

Similar to that conducted by Lumbreras and his colleagues,^[11]^ all studies were classified into three categories in terms of their clinical significance: type I, type II, and type III. Type I refers to highly clinically-significant findings that required further imaging follow-up or immediate therapeutic intervention, including malignancy or curable tumor, pulmonary embolism, and intracranial aneurysms. Type II was defined as moderately clinically-significant findings that required no immediate action but necessary follow-up, including enlarged lymph nodes, thyroid lesions, sinusitis, and ground-glass opacities. Type III was designated as minor or no significant findings that required no follow-up, such as degeneration of spine, arachnoid cyst, and atelectasis, and were excluded from this study.

Type I and type II IFs were regarded as potential clinically significant findings and assessed in our study. Another two radiologists with 10 and 26 years of experience retrospectively reviewed each “potential clinically significant IF” case seen on the neck CTA images, respectively. If both radiologists agreed with the initial radiologist, it was classified as potential clinically significant IF (Kappa value = 0.813). Any discrepancies in extracted data were discussed, and disagreements were resolved by consensus with a fourth radiologist with 21 years of experience resulting in a diagnostic agreement of IFs. All IFs were recorded and compared with the original report.

### 2.4 Statistical analysis

All data were analyzed using the Statistical Package for the Social Sciences (SPSS, version 16.0; SPSS, Inc., Chicago, IL, USA). Discrete and continuous data were reported as counts and percentages, and as mean ± SD, respectively. Comparisons of categorical variables were performed and Chi-square test was used when appropriate. A *P* value of less than .05 was considered statistically significant.

## 3 Results

A total of 1862 consecutively-registered cases underwent neck CTA during the 2-year study period, of which 135 (7.3%) cases were excluded due to artifacts, 2 (0.1%) due to irretrievable images, and 27 (1.5%) for repeated CTAs. The remaining 1698 cases consisted of 922 males and 766 females with an average age of 54.7 ± 10.6 years (age range: 34–86 years).

A total of 376 potential clinically significant IFs were identified in 355 (20.91%) patients. The rest 1,343 (79.09%) patients had type III or no IFs and were thus not included in this study. Of the 376 IFs, 159 (42.29%) were found in the chest and 217 (57.71%) in the head or neck; 175 (46.54%) were regarded as type I and 201 (53.46%) as type II. The most common identified potential significant IFs were thyroid nodules (n = 88, 23.40%), pulmonary nodules (n = 56, 14.89%), sinus disease (n = 39, 10.37%), intracranial or cervical artery aneurysms (n = 30, 7.98%), enlarged lymph nodes (n = 24, 6.38%), and pulmonary embolism (n = 19, 5.05%). Other IFs were rare. Among these 376 IFs, 184 (48.94%) were not mentioned in initial CTA reports. The unreported proportion of chest IFs was 73.58% (117/159), significantly higher than that of head or neck IFs [30.88% (67/217); χ2 = 65.286, *P* < .001, Chi-square test].

**Figure 1:**
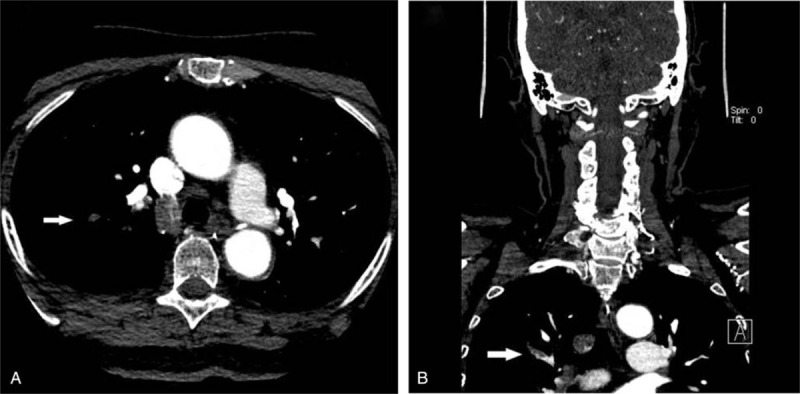
Axial (A) and coronal (B) CTA images demonstrating a pulmonary embolism within right upper lobe pulmonary artery (white arrow). This incidental finding was not initially reported on neck CTA.

**Figure 2:**
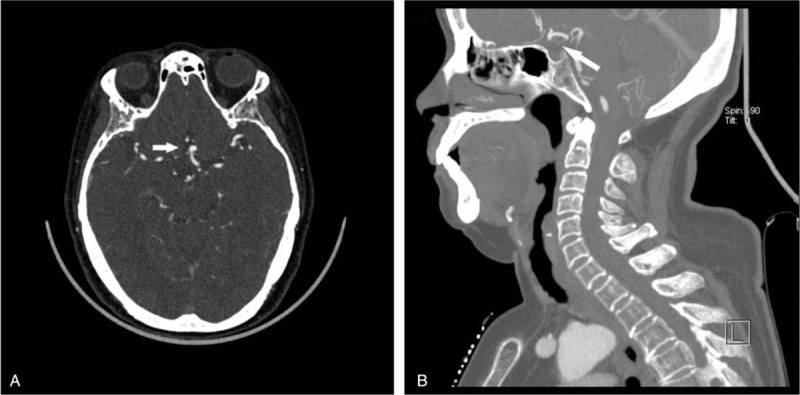
Axial (A) and sagittal (B) CTA images demonstrating an intracranial artery aneurysm of the anterior communicating artery (white arrow). This incidental finding was not initially reported on neck CTA.

**Figure 3:**
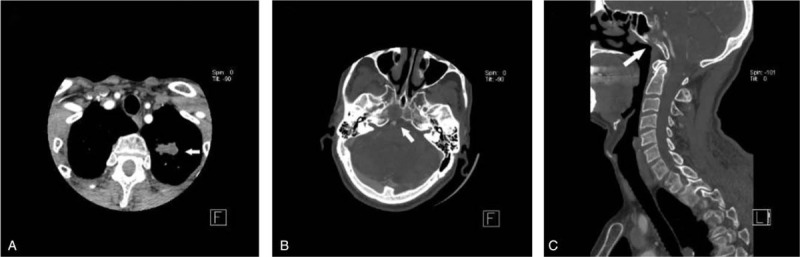
Axial (A) CTA image demonstrating a 1.7 cm pulmonary nodule in upper lobe of the left lung (white arrow). This incidental finding was initially mentioned on neck CTA. Axial (B) and sagittal (C) CTA images demonstrating a bony destruction of sphenoid bone (white arrow), which was, inversely, not initially reported.

The highly clinically significant findings were summarized in Table 1. The IF incidence ranged from as high as 17.71% in the group with intracranial/cervical artery aneurysms (n = 30), 14.29% in the group with thyroid nodule (>2 cm) (n = 25), 13.71% in the group with enlarged lymph nodes (n = 24), 10.86% in the group with pulmonary embolism (n = 19), to 9.17% in the group with lung nodule (> 1 cm) (n = 16). Other relevant findings were scarce. The unreported rate of incidental findings was as follows: all pulmonary embolism (19/19 patients, 100%) and pathologic fractures and erosions (4/4 patients, 100%) were not mentioned in the initial CT reports, followed by esophageal wall thickening (5/6 patients, 83.33%), arterial aneurysm (8/10 patients, 80%), and acute pneumonia (6/8 patients, 64%). Only one case of large pleural effusions and sinus solid mass was mentioned in the initial CTA report. Of all the highly clinically significant IFs, only 46.86% were mentioned in the initial written reports. In terms of anatomical classification, 60 and 33 unmentioned IFs were respectively found in chest and head or neck, with a respective incidence rate of 77.97% (60/77) and 33.67% (33/98). Thus, the incidence of unreported Type I IFs in the chest group was significantly higher than in the head and neck one (χ^2^ = 32.151, *P* < .001, Chi-square test). Examples of Type I IFs are provided in Figures 1–3.

**Table 1:**
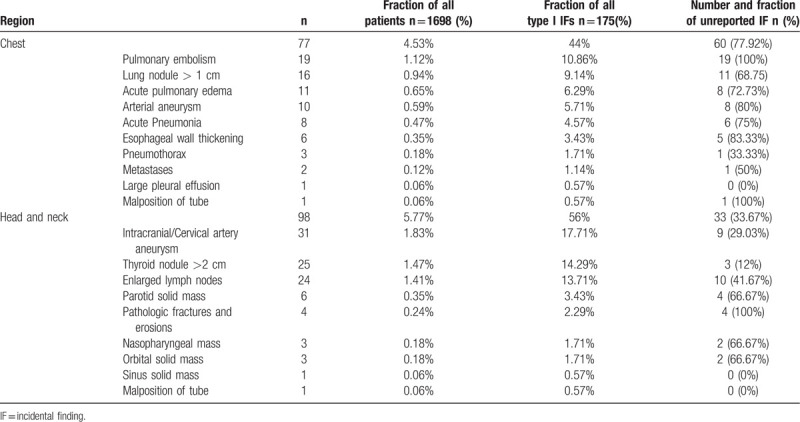
Summary of type I findings of highly clinical significance.

**Figure 4:**
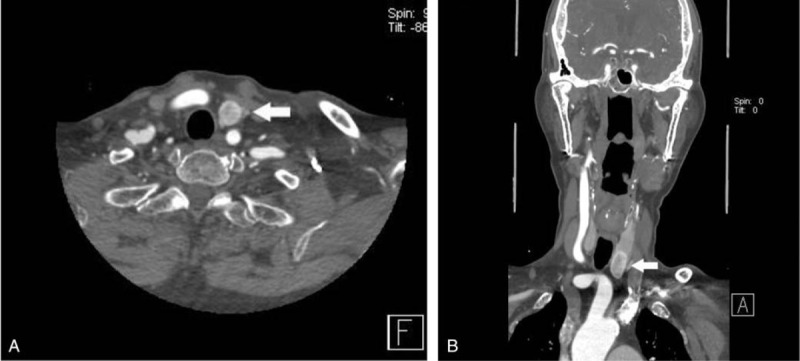
Axial (A) and coronal (B) CTA images demonstrating a 1.3 cm thyroid nodule within the left lobe of the thyroid gland (white arrow). This incidental finding was not initially reported on neck CTA.

**Figure 5:**
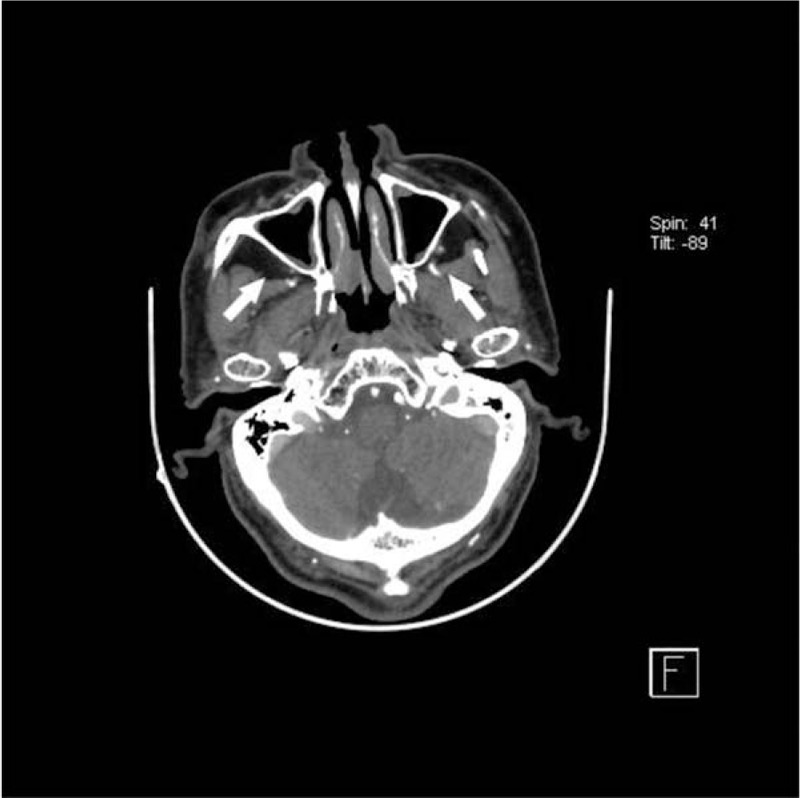
Axial (A) CTA image demonstrating inflammation in both maxillary sinuses (white arrow). This incidental finding was initially reported on neck CTA.

For the moderately clinically-significant group, the detailed distribution of IFs were outlined in Table 2. The most common was 63 cases of thyroid nodule (≤ 2 cm – > 1 cm, 31.34% in this group), followed by 40 cases of lung nodule (≤ 1 cm – > 0.4 cm, 19.90%), 39 cases of sinus disease (19.40%), 22 cases of pleural plaque (10.95%) and 17 cases of small pleural effusion (2.43%). Other kinds of IFs were all smaller than 10 cases. Of all Type II IFs, 1 case of vocal cord nodule (1/1, 100%) was unmentioned by the initial radiologist, followed by 29 cases of lung nodule (≤ 1 cm – >0.4 cm) (29/40, 72.5%), 15 cases of pleural plaque (15/22, 68.18%), 2 cases of pulmonary artery dilatation (2/3, 66.67%) and 11 cases of small pleural effusion (11/17, 64.71%). A total of 7 cases of soft-tissue cystic mass (7/9, 77.78%) were recorded in the diagnostic reports. The unreported incidence rate in the chest group and head or neck group was 69.51% (57/82) and 28.57% (34/119), respectively. Thus, the incidence of unreported type I IFs in the chest group was significantly higher than in the head and neck one (χ^2^ = 31.211, *P* < .001, Chi-square test). Examples of type II IFs are shown in Figures 4 and 5.

**Table 2:**
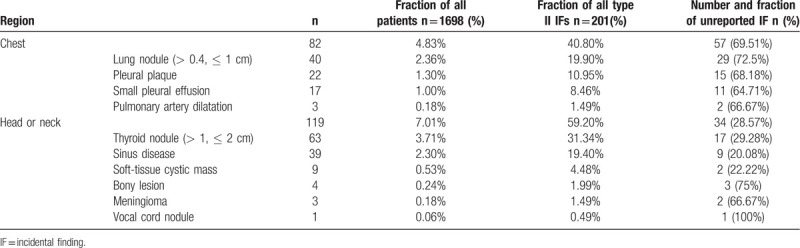
Summary of type II findings of moderately clinical significance.

## 4 Discussion

Computed tomographic angiography is not uncommon for noninvasive evaluation of patients with neck or head diseases. As the field of view in CTA examinations includes some additional organs, such as the pulmonary parenchyma or soft tissue structures, it is vital to inspect all those structures for potential diseases. The present study investigated the frequency, distribution and unreported rate of potential clinically significant IFs on neck CTA images. To our best knowledge, this is the first study focusing on evaluating IFs on neck CTA scans from a large non-selected cohort.

A total of 376 potential clinically significant IFs were identified in 20.91% of the patients, of which 46.54% of IFs were considered as highly clinically significant incidental findings and 53.46% as moderately clinically significant. The rate of clinically significant IFs in our study was not different from that of a previous similar study, which identified actionable, incidental findings in 21.5% of patients receiving acute stroke intervention combined with CTA of the head and neck.^[2]^ This might be expected, as we found some IFs, that is, pulmonary embolism, which were not reported before, even though our data of neck CTA cases have smaller coverage and fewer images.

Thyroid nodule is one of the most familiar IFs on imaging studies that involve the neck. In our study, the prevalence of thyroid nodule (>1 cm) was 5.18%, representing the most common of potential clinically significant IFs. This is lower than the reported average incidence from CT scans targeting the region of neck from the literature,^[12,13]^ but is comparable to that in patients undergoing CT cervico-cerebral angiography.^[14]^ The low incidence of thyroid nodule may result from the inclusion criterion set in the current study, in which we only included thyroid nodule (>1 cm) into our research, for the previous study shows that failure to diagnose thyroid cancers (≤1 cm) is unlikely to influence morbidity and mortality.^[15]^

Of note, in the current study, the prevalence of pulmonary embolism in our study was 1.12%. Pulmonary embolism has not been described as IF in CT imaging of the head and neck CTA examinations specialized in IFs.^[6,14]^ However, pulmonary embolism has been identified in 0.8% to 7.6% studies by other different research methods.^[16,17]^ Therefore, the incidence of pulmonary embolism in patients undergoing neck CTA examination is probably underestimated, as neck CTA only involves a limited part of the upper portion of pulmonary artery but the lower lung artery is the predilection site of pulmonary embolism.^[18]^ The other possible cause is that not all the arteries in neck CTA images meet the appraisal criteria of pulmonary embolism.

The unreported rate of potential clinically significant IFs in the primary report was 48.92% (73.58% and 30.88% in chest and head or neck, respectively), which is lower than that of a study of chest imaging. The latter found that potential significant cardiovascular IFs were unreported in 63.2% of patients undergoing non-ECG-synchronized chest CT examinations.^[19]^ The observed high unreported rate may be attributed to the following factors: firstly, a phenomenon known as “inattentional blindness” may contribute to under-reporting.^[20]^ CT is usually performed to find the actual cause of a known disease or symptom. Therefore, radiologists often focus more on the clinical interest than on the potentially more clinically significant IFs. Another phenomenon called “satisfaction of search” may also be attributable when a lesion to answer a clinical question was found, leading to the termination of evaluation of other abnormalities, because the radiologist is contented with the detected findings.^[21]^ A third reason may be due to too much work and not enough manpower in our hospital.

As seen, the reported rate of IFs in the head or neck group was higher than that of the chest group. Several factors may account for the relatively higher rate of under-reporting of chest findings. The most important one is that the suitable CT window and level for evaluating lung was not provided in previous images. The other possible reason is a lack of sufficient training and low confidence on the part of the initial reporting radiologists in identifying chest IFs on standard neck CTA.

Thyroid nodule accounted for a large fraction of potential clinically significant IFs (23.40%), but most of these (68/88) were reported by initial radiologists. Thyroid nodules are almost of low density and can easily be detected in neck CT, because normal thyroid gland contains iodine and presents a high attenuation even on non-contrast CT.^[22]^ On the contrary, all incidental pulmonary embolisms were missed in the initial report, though they were identified in 19 (1.12%) patients. One possible cause is that radiologists are inclined to concentrate on carotid arteries rather than on pulmonary vessels. Meanwhile, the diagnosis of PE on neck CTA scans seems more difficult than that on standard pulmonary CTA images, for the conspicuity of pulmonary vessels is not comparable to that from pulmonary CTA.

Several possible limitations are present in this study. Firstly, it was a single-center study, and the regional patient population may have pathogenic factors and diseases different from those of other regions. Thus, the results may not be readily replicated to all hospitals, and their clinical significance may be limited. Secondly, as a retrospective study, the follow-up data of all patients with IFs were not available, so all IFs were not subjected to further investigations and follow-up. The discovery of some IFs (eg, pulmonary embolism) are largely dependent on the radiologist's professional experience. However, the IFs diagnostic criteria used in the current study are relatively strict. Thirdly, the use of arterial phase imaging in the examination of the neck, head and upper thorax may limit the evaluation of hypo-vascular lesions and pathology, which may be more reliably detected in a delayed phase. Moreover, we did not consider whether the incidental findings were already known to the clinicians. However, we assume that this did not substantially influence the reporting rate of the IFs, for clinical information on these diseases is seldom provided to radiologists before reporting.

## 5 Conclusion

The current paper reveals the high prevalence of potentially life-threatening incidental findings in unselected patient population undergoing neck CTA and exposes that nearly half of these IFs, especially those in the chest region, are not mentioned in the original report. The results signify that due care should be given to incidental findings during neck CTA. To reduce the missed rate of incidental findings, a definite categorization of incidental findings in neck CTA is necessary for interpreting significance of such findings. Moreover, the clinicians should provide sufficient clinical information of the patients and radiologists should endeavor to access as detailed data of the patients as possible before the imaging examination.

## Author contributions

**Conceptualization:** Guang-Liang Chen, Yun-jing Xue, Qing Duan.

**Data curation:** Guang-Liang Chen, Yun-jing Xue, Jin Wei.

**Formal analysis:** Guang-Liang Chen, Yun-jing Xue.

**Investigation:** Guang-Liang Chen.

**Methodology:** Guang-Liang Chen, Yun-jing Xue, Jin Wei, Qing Duan.

**Project administration:** Guang-Liang Chen, Yun-jing Xue.

**Resources:** Jin Wei, Qing Duan.

**Software:** Jin Wei.

**Supervision:** Yun-jing Xue, Qing Duan.

**Validation:** Guang-Liang Chen, Yun-jing Xue.

**Visualization:** Guang-Liang Chen.

**Writing – original draft:** Guang-Liang Chen, Yun-jing Xue.

**Writing – review & editing:** Guang-Liang Chen, Yun-jing Xue.
